# Artificial Intelligence for Computer-Aided Detection in Endovascular Interventions: Clinical Applications, Validation, and Translational Perspectives

**DOI:** 10.3390/bioengineering13040399

**Published:** 2026-03-29

**Authors:** Rasit Dinc, Nurittin Ardic

**Affiliations:** 1 INVAMED Medical Innovation Institute, One World Trade Center, 85th Floor, 285 Fulton Street, New York, NY 10007, USA; 2 Med-International UK Health Agency Ltd., 56 Pallett Dr, Nuneaton CV11 6LT, Warwickshire, UK; nurittinardic@yahoo.com

**Keywords:** artificial intelligence, computer-aided detection, endovascular interventions, validation, regulatory science, endovascular imaging

## Abstract

Background: Artificial intelligence-based computer-aided detection (AI-CAD) systems are increasingly being used in endovascular practice to support time-sensitive detection, triage and prioritization tasks in imaging and procedural workflows. Despite rapid technological advancements and expanding regulatory clearances, the translation to lasting clinical benefit varies. Objective: This narrative review synthesizes AI-CAD applications in endovascular interventions and proposes an evaluation-oriented framework to support responsible clinical translation; this framework emphasizes detection-specific metrics, external validation, bias-aware assessment, and workflow integration. Methods: A structured narrative review was conducted using targeted searches in PubMed, Google Scholar, and IEEE Xplore (2020–2026); this review was supported by an examination of US FDA device databases and citation tracking. Evidence was assessed using a pragmatic hierarchical classification framework based on regulatory status and validation rigor. Results: AI-CAD applications were mapped across four main endovascular domains: neurovascular interventions (e.g., large vessel occlusion triage), coronary interventions (CCTA-based stenosis detection and intravascular imaging support), aortic interventions/EVAR (endoleak detection and sac monitoring), and peripheral interventions (lesion detection and angiographic decision support). Across the domains, performance reporting was heterogeneous and often relied on retrospective, single-center assessments. Key barriers to clinical readiness included acquisition variability and dataset shift due to artifacts, limited multicenter validation, annotation variability, and human–AI workflow factors. Evaluation priorities included whether to assess at the lesion level or case level, false positive burden and calibration, external validation under real-world heterogeneity, and clinical impact measures such as treatment timing and procedural decision-making. Conclusions: AI-CAD systems hold significant potential for improving endovascular care; however, clinical readiness depends on rigorous, endovascular feature-specific assessment and transparent reporting, beyond retrospective accuracy. The proposed evidence level framework and assessment checklist provide practical tools for distinguishing mature technologies from research prototypes and guiding future validation, implementation, and post-market monitoring.

## 1. Introduction

Cardiovascular diseases (CVDs) remain the leading cause of mortality worldwide, accounting for approximately 19.8 million deaths in 2022 [1]. Despite transformative advances in endovascular catheter-based interventions over the past three decades, accurate interpretation of increasingly complex multimodal imaging remains a significant clinical challenge.

Interpretation of complex multimodal imaging data, including computed tomography angiography (CTA), digital subtraction angiography (DSA), magnetic resonance angiography (MRA), and intravascular ultrasound (IVUS), requires specialized expertise that is not uniformly available across healthcare settings [2]. Diagnostic errors remain a significant concern for patient safety. In outpatient settings, estimates suggest that approximately 1 in 20 adults in the U.S. experience a diagnostic error each year, with a significant proportion potentially associated with harm [3,4]. In emergency care, potential diagnostic errors have also been linked to worse outcomes, such as higher 30-day mortality rates and reduced healthy days spent at home [5]. In acute ischemic stroke, an estimated 1.9 million neurons are lost every minute of untreated large vessel occlusion [6]. These observations highlight the critical need for rapid and accurate diagnostic tools.

Artificial intelligence (AI) has rapidly transitioned from experimental research to clinical application in many branches of medicine, including medical imaging [7]. In cardiovascular and endovascular applications, AI-enabled systems are increasingly integrated into imaging analysis, emergency triage, and procedural workflows. Regulatory transparency has also improved, with publicly available databases documenting a growing number of AI-based medical devices cleared for clinical use [8]. The convergence of increasing procedural complexity and rapidly advancing AI has created new opportunities for imaging-assisted decision support while simultaneously introducing significant translational challenges.

Within this evolving landscape, computer-aided detection (CAD) has emerged as a clinically important class of AI applications designed to identify actionable abnormalities in imaging and procedural data streams. CAD systems are designed to automatically identify clinically relevant abnormalities such as large vessel occlusion, hemodynamically significant coronary stenosis, aneurysm-related complications, or endoleaks, thus assisting clinicians in time-sensitive decision-making. Unlike long-term predictive modeling tools, CAD systems primarily focus on detection and triage in imaging or procedural data streams, while segmentation and quantitative analysis modules often support detection workflows.

**Figure 1:**
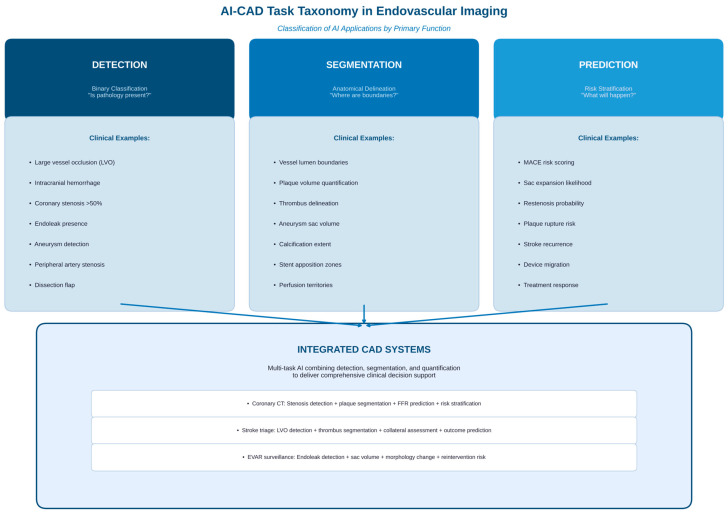
AI-CAD task taxonomy in endovascular imaging. This figure classifies AI applications in endovascular practice according to their primary clinical functions. Three distinct categories are shown: (1) detection tasks focus on binary classification to determine the presence of pathology, including major vessel occlusion, intracranial hemorrhage, coronary stenosis, endoleaks, and aneurysms; (2) segmentation tasks identify anatomical boundaries such as vessel lumen, plaque volumes, thrombus spread, and calcification distribution; and (3) prediction tasks classify risk and predict outcomes, including major adverse cardiac events (MACE), sac expansion, probability of restenosis, and treatment response. The sub-section illustrates integrated CAD systems that combine multiple tasks; for example, coronary CT analysis platforms that simultaneously perform stenosis detection, plaque segmentation, fractional flow reserve (FFR) estimation, and cardiovascular risk classification. These multitasking systems represent an evolution toward comprehensive clinical decision support platforms that utilize detection, segmentation, and prediction in unified workflows.

To contextualize the scope of AI applications discussed in this review, Figure 1 illustrates the conceptual taxonomy of AI-CAD tasks in endovascular imaging and distinguishes detection-oriented systems from segmentation and prediction-oriented approaches.

Advances in deep learning, including convolutional neural networks and transformer-based architectures, have significantly improved automated detection capabilities in medical imaging [9]. Parallel to these technical advancements, analyses of regulatory clearances show a marked increase in FDA-cleared AI/machine learning-powered devices in recent years [10]. Cardiovascular applications similarly show increasing representation among regulatory-cleared AI systems, with most devices validated through established regulatory pathways [7,11]. However, regulatory clearance, by itself, does not guarantee robust external validation or consistent real-world performance.

In endovascular medicine, the integration of AI-based CAD (AI-CAD) tools presents both opportunities and challenges. Imaging variability, contrast timing differences, metal artifacts from implanted devices, and inter-institutional protocol heterogeneity can significantly impact detection performance. Furthermore, evaluation methodologies in published studies remain heterogeneous, with inconsistent reporting of detection-specific metrics and limited external validation. Such variability complicates inter-study comparisons and may mask limitations only revealed during real-world application.

Emerging evidence suggests that detection-oriented AI systems can significantly impact clinical workflows when integrated into acute care pathways. For example, automated large vessel occlusion detection software has been associated with improvements in treatment duration metrics in stroke networks [12,13]. Additionally, recent advances demonstrate the applicability of intra-procedural AI detection systems, such as deep learning models for automated endoleak detection during digital subtraction angiography in endovascular aneurysm repair [14]. These examples illustrate the increasing integration of AI-CAD systems into real-time interventional decision-making.

Despite the rapid growth of AI-CAD systems in cardiovascular imaging, their translation to endovascular applications requires rigorous evaluation, as both false negative and false positive detections can have significant clinical consequences in time-sensitive interventional workflows. However, the current literature reveals several significant gaps that limit their translation to endovascular applications. First, most existing reviews focus on technical performance or modality-specific applications rather than the clinical translation pathway of AI systems in endovascular workflows [9,10]. Second, there is currently no domain-specific evaluation framework that integrates detection-focused performance metrics, regulatory status, and clinical validation evidence tailored to the unique characteristics of endovascular imaging environments. Third, performance reporting remains heterogeneous across studies [15,16], with inconsistent reporting of lesion-level versus case-level metrics, limited multicenter validation, and insufficient attention to dataset shifts arising from metallic artifacts and procedural imaging dynamics. These limitations make it difficult for clinicians, bioengineers, and regulatory stakeholders to determine which AI-CAD diagnostic systems are mature enough for real-world clinical integration. In particular, the absence of a structured hierarchy of evidence and standardized evaluation criteria complicates comparisons between early research prototypes and regulatory-approved technologies.

To address these challenges, this review presents a clinically focused synthesis of AI-CAD applications in endovascular interventions and proposes a structured framework to support translational evaluation. Specifically, the objectives of this review are:

(1) To synthesize current AI-CAD applications in major endovascular domains, including neurovascular, coronary, aortic (EVAR), and peripheral interventions.
(2) To propose a pragmatic evidence classification framework that distinguishes regulatory-approved systems with independent validation from emerging research prototypes.
(3) To identify key methodological considerations for evaluation and validation, including detection-specific metrics, external validation strategies, bias assessment, and workflow integration.
(4) To outline translational challenges and future research priorities for the safe and effective implementation of AI-CAD systems in heterogeneous endovascular imaging settings.

By integrating technical, clinical, and regulatory perspectives, this review provides a structured reference for evaluating AI-CAD systems and introduces a pragmatic evidence classification framework and evaluation checklist aimed at supporting the translation of AI technologies into endovascular practice.

## 2. Materials and Methods

### 2.1. Review Design

This study was conducted as a structured narrative review to synthesize current knowledge about AI applications in CAD within cardiovascular and endovascular medicine. Unlike systematic or scoping reviews, this study does not aim to conduct extensive study capture or quantitative meta-analysis. Instead, it presents an expert-focused synthesis of clinically relevant and methodologically significant advances in endovascular AI-CAD systems.

The scope of this review primarily focuses on detection- and triage-oriented AI systems applied to endovascular imaging and procedural workflows. Segmentation and quantitative analysis tools were included where they directly support detection tasks or influence real-time clinical decision-making processes.

### 2.2. Literature Identification and Selection

Relevant literature was identified through targeted searches in PubMed, Google Scholar, and IEEE Xplore covering the period from January 2020 to January 2026. Search terms included combinations of “artificial intelligence,” “deep learning,” “computer-aided detection (CAD),” “cardiovascular imaging,” “coronary artery disease,” “endovascular,” “aortic aneurysm,” “endoleak detection,” and “intravascular imaging.”

In addition to database searches, we reviewed the U.S. Food and Drug Administration (FDA) 510(k), De Novo, and pre-market approval (PMA) databases to identify AI-enabled cardiovascular and endovascular devices with regulatory clearance. Where available, publicly available CE mark documentation was also considered. To ensure the inclusion of effective and widely adopted systems, citation tracking of key publications and reference lists of significant review articles were performed. Significant clinical trials were identified based on both literature searches and the authors’ clinical and research expertise.

Because this review represents a narrative synthesis rather than a systematic review, formal PRISMA-based study screening and record quantification were not performed.

### 2.3. Evidence Classification Framework

Given the heterogeneity of the available evidence, we developed a pragmatic tiered classification framework (Table 1) to categorize AI-CAD systems according to regulatory status and validation rigor. Tier 1A includes regulatory-cleared systems supported by peer-reviewed clinical validation and independent evaluation. Tier 1B includes regulatory-cleared systems where performance data is primarily reported by the manufacturer. In early clinical deployment, Tier 1B systems should be interpreted more cautiously than Tier 1A systems, as manufacturer-reported performance may not fully reflect robustness under independent real-world conditions. In practice, while regulatory approval supports the defined intended use, local adoption decisions should also consider external validation, workflow compliance, and post-implementation monitoring [8,10,11]. Tier 2 encompasses peer-reviewed research systems without regulatory clearance. Tier 3 includes industry-reported systems or conference abstracts without full peer-reviewed validation, and Tier 4 represents technologies in the conceptual or developmental stage.

This framework makes it possible to differentiate between clinically mature technologies and emerging research prototypes while maintaining transparency regarding the strength of evidence.

### 2.4. Methodological Limitations

As a narrative review, this synthesis reflects expert interpretation of selected literature rather than a comprehensive systematic search. Although efforts have been made to prioritize regulatory-cleared systems with peer-reviewed clinical trials and published validation data, relevant studies may not have been comprehensively captured. Furthermore, heterogeneity in study design, dataset composition, and performance reporting limits direct comparability between systems.

## 3. Clinical Applications of AI-CAD in Endovascular Interventions

AI-CAD systems have been developed across multiple endovascular domains, targeting time-sensitive diagnostic tasks and intra-procedural imaging challenges. While the maturity of these systems varies depending on the clinical context, detection-oriented applications have gained momentum, particularly in neurovascular, coronary, aortic, and peripheral vascular interventions. This section summarizes representative clinical applications, highlighting detection functionality, validation status, and translational relevance. To contextualize clinical applications in vascular fields, Table 2 summarizes representative AI-CAD systems in terms of primary task, imaging method, regulatory status, and evidence tier.

### 3.1. Neurovascular Interventions

Neurovascular intervention represents one of the most advanced and clinically integrated areas for AI-CAD systems. Rapid detection of large vessel occlusion (LVO) in acute ischemic stroke is critical for timely referral to mechanical thrombectomy, as neuronal loss progresses rapidly in untreated occlusion [6]. Automated LVO detection algorithms applied to CTA have therefore become a central example of clinically used AI-CAD systems.

Various AI systems available on the market utilize deep convolutional neural networks to detect occlusions in segments of the internal carotid artery or proximal middle cerebral artery. Clinical evaluation studies have shown that the integration of automated LVO detection software into stroke networks can improve workflow efficiency. In a cluster randomized clinical trial, the implementation of AI-assisted LVO detection was associated with a reduction in treatment delays [12]. Similarly, machine learning-assisted automated LVO detection has been associated with improved transfer times from primary stroke centers to comprehensive stroke centers [13]. These findings suggest that, in this context, the fundamental benefit of AI-enabled decision support may stem not only from diagnostic performance metrics but also from accelerated team notification and workflow coordination. However, the clinical benefit of automated triage also depends on its false positive burden. Although a universal threshold for acceptable alert frequency has not been established, excessive false positive reporting, particularly in high-volume stroke networks [12,13,17], can contribute to alert fatigue, reduce clinician confidence, and decrease workflow utility.

Beyond LVO detection, AI-based CAD tools have been developed for the identification of intracranial hemorrhage on non-contrast CT and cerebral aneurysm detection with CT angiography (CTA) and magnetic resonance angiography (MRA). While retrospective validation studies frequently report high sensitivity and area under the receiver operating characteristic curve (AUROC) values, performance variability remains affected by lesion size, anatomical location, image quality, and motion artifacts. Furthermore, algorithm performance can differ between institutions due to scanner heterogeneity and contrast timing protocols, highlighting the importance of external validation.

Neurovascular AI-CAD systems also illustrate the distinction between regulatory clearance and clinical effectiveness. Analyses of FDA-cleared AI/machine learning-assisted medical devices show rapid growth in radiology-based AI systems in recent years [10]. However, regulatory clearance primarily determines substantial equivalence or safety within defined indications and does not guarantee consistent real-world performance across diverse clinical settings. In conclusion, future workflow integration studies, multicenter validation cohorts, and post-implementation monitoring are critical for achieving sustainable clinical benefit.

Overall, neurovascular applications offer a model for understanding how detection accuracy, system integration, and healthcare system organization interact to determine the real-world impact of AI-CAD systems in endovascular application.

### 3.2. Coronary Interventions

Coronary artery disease remains a leading cause of morbidity and mortality and presents a significant area for developing AI-CAD in both non-invasive and invasive imaging pathways. Unlike neurovascular stroke triage, where detection is often framed as an emergency alert task, coronary AI-CAD encompasses (i) anatomical detection of stenosis and plaque phenotypes in coronary computed tomography angiography (CCTA) and (ii) catheter-based intravascular imaging interpretation during percutaneous coronary intervention (PCI), including intravascular ultrasound (IVUS) and optical coherence tomography (OCT). These applications reflect a fundamental bioengineering theme: engineering methods that translate high-dimensional imaging into clinically viable detection outputs. Validation requirements also differ between physiology-based platforms and binary detection tools. While binary detection systems are primarily evaluated based on their discrimination and localization performance, physiology-based models such as FFR-CT also need to be assessed for their agreement with reference physiological measurements, the robustness of their computational assumptions, and consistency across varying image quality and anatomical complexity [18,19].

#### 3.2.1. CCTA-Based Detection of Coronary Stenosis

Automated detection and grading of coronary stenosis using CCTA is among the most widely researched coronary AI-CAD applications. Contemporary deep learning systems typically combine vessel extraction, centerline tracking, and lumen analysis to localize stenotic segments and provide severity estimates. Recent multicenter studies have demonstrated the feasibility of deep learning-assisted CCTA analysis for large-scale quantification of stenosis and plaque-related features, highlighting the potential of automated workflows to improve reproducibility and efficiency in coronary imaging [20]. In parallel, clinical trials have reported that AI-based interpretation of CCTA can approach expert-level performance in identifying clinically significant coronary disease in compiled datasets, supporting its role as a detection-oriented triage tool in diagnostic pathways [18,19].

However, CCTA-based detection performance is sensitive to image quality, motion, dense calcification, and differences in scanner hardware and reconstruction protocols. These factors can lead to dataset shift between institutions and reduce generalizability if algorithms are only validated internally. Accordingly, transparent reporting of external validation and acquisition conditions is essential, especially when AI outputs are used to guide subsequent invasive testing or procedure planning.

#### 3.2.2. Plaque Characterization as Detection Enhancement

Beyond detecting lumen narrowing, AI methods are increasingly aiming to identify plaque phenotypes and high-risk imaging signatures that can influence intervention strategy. From a coronary disease perspective, plaque characterization functions as a feature-level detection tool: the algorithm identifies patterns such as calcified and non-calcified plaque, adverse remodeling features, or other morphological signatures that could alter procedural complexity or risk. Multicenter CCTA studies incorporating deep learning have highlighted the automated extraction of plaque/stenosis descriptors as a scalable approach for structured coronary assessment [20]. Additionally, automated or semi-automated systems have been proposed to assess stenosis and plaque-related features together in CCTA, although the clinical impact of these tools is still being actively evaluated [21].

Importantly, while plaque phenotyping can enrich risk stratification, much of the evidence supporting these approaches is retrospective, and demonstrating outcome utility at the patient level often requires prospective evaluation and careful integration into the clinical decision-making process. Therefore, in the context of this review, plaque characterization is considered an enabling component of CAD rather than a definitive prognostic tool.

#### 3.2.3. AI-CAD in Intravascular Imaging (IVUS and OCT)

Intravascular imaging provides high-resolution assessment of plaque morphology and stent placement and is routinely used for optimizing percutaneous coronary intervention (PCI) in complex lesions. OCT, in particular, allows for near-microscopic visualization of the vessel lumen and stent struts, creating an opportunity for AI-based tools to reduce analysis time and inter-observer variability. Automated lumen segmentation in intracoronary OCT has been demonstrated using fully automated approaches, supporting rapid quantitative assessment during procedural workflows [22]. Beyond lumen segmentation, AI methods have been applied to plaque tissue characterization in OCT, including automated classification frameworks aimed at identifying morphological plaque components and guiding intra-procedural interpretation [23]. It has been reported that deep learning-based segmentation has also been used for specific lesion subtypes, such as calcified plaque in intravascular OCT, demonstrating the applicability of detection/segmentation modules tailored to interventional questions [24].

Despite these advances, intravascular AI-CAD systems face various engineering challenges, including catheter motion artifacts, blood attenuation, shadowing due to calcification, and variability in retraction acquisition parameters. Additionally, training datasets for IVUS/OCT are generally smaller and less standardized than CCTA datasets, which can limit model generalization. These limitations reinforce the importance of careful consideration of multicenter validation, standardized annotation protocols, and how AI outputs interact with real-world operator decision-making processes in catheterization labs.

Collectively, coronary applications demonstrate the expanding impact of AI-CAD in both non-invasive and catheter-based imaging. While automated detection performance continues to improve, clinical readiness relies on robust external validation, consistent measurement reporting, and evidence that AI-powered detection outputs significantly improve workflow efficiency and procedural decision-making in real clinical settings.

### 3.3. Aortic and Endovascular Aneurysm Repair (EVAR)

Endovascular aneurysm repair (EVAR) is a technically challenging field where imaging interpretation directly impacts procedural success and long-term follow-up. Post-EVAR complications (particularly endoleaks, sac enlargement, graft migration, and device-related failures) require accurate detection on multimodal imaging platforms, with CTA remaining the primary surveillance modality [25]. In this context, AI-CAD systems aim to automate complication identification, improve reproducibility, and reduce interpretive variability during longitudinal follow-up.

#### 3.3.1. Endoleak Detection on CTA

Endoleak detection based on CT angiography has become an active area in artificial intelligence development. Deep learning approaches have been applied to multiphase CT angiography datasets for the automatic identification and localization of endoleaks, and their applicability has been demonstrated in retrospective validation cohorts [14]. In this environment, detection performance is affected by factors such as contrast timing variability, metallic artifacts from stent grafts, motion artifacts, and aneurysm sac morphology; these factors are known to make endoleak interpretation difficult, even among expert readers [25].

While automated detection can support structured reporting and longitudinal sac tracking, most studies are retrospective and single-center. External validation across institutions and imaging platforms is still limited, highlighting the need for rigorous multi-center evaluation before widespread clinical integration.

#### 3.3.2. Intra-Procedural Endoleak Detection on DSA

Recent advances have extended AI-CAD technology into the intra-procedural realm. Multitasking deep learning models have been developed for automated endoleak detection during DSA performed during EVAR [14]. This shift from post-procedural surveillance imaging to real-time procedural support represents a significant translational step.

From a bioengineering perspective, intra-procedural DSA detection introduces additional complexities, including dynamic contrast flow, variable frame acquisition, catheter motion artifacts, and real-time computational constraints. For intraprocedural use, latency must be low enough to preserve the relevance of the procedural relevance during completion angiography. Although standardized thresholds have not yet been established, clinically useful systems will likely require near real-time or sub-minute output so that alerts can influence immediate decision-making before catheter removal or procedure completion [14,17]. The feasibility demonstrated in early validation studies suggests potential for integration into procedural workflows, but prospective outcome-based validation is still necessary.

#### 3.3.3. Sac Expansion and Morphological Monitoring

Beyond binary endoleak detection, volumetric monitoring of aneurysm sac size is a central component of post-EVAR follow-up. Sac expansion is a known indicator of persistent endoleak or device-related failure and is routinely assessed during longitudinal follow-up [25]. AI-based segmentation algorithms applied to CTA have been investigated to automate aneurysm sac segmentation and facilitate reproducible volume tracking over time [2]. In this context, AI-assisted 3D volumetric segmentation should not be considered equivalent to manual 2D diameter measurement. While diameter-based assessment is simpler and more clinically familiar, it can be more sensitive to slice selection and geometric assumptions; volumetric approaches, on the other hand, can better capture asymmetric sac remodeling, but also bring their own sources of segmentation error and dependence on image quality [2,25].

Such segmentation-driven detection workflows demonstrate the integration of engineering modules into CAD pipelines: automated segmentation enables secondary detection tasks such as identifying abnormal sac growth trends. However, segmentation ambiguity, inter-scan variability, and metallic artifacts remain technical challenges that can impact reliability in real-world practice.

#### 3.3.4. Translational Considerations in Aortic AI-CAD

Compared to neurovascular large vessel occlusion (LVO) detection systems, aortic AI-CAD tools are at an earlier stage in the translational pathway. Regulatory analyses show significant growth in AI-assisted radiological devices in general [10], but the number of systems specifically targeting the detection of EVAR complications is relatively small. Consequently, many aortic AI-CAD systems currently fall into Tier 2 or Tier 3 categories within the evidence framework described in Section 2.

Demonstrating the long-term benefit in EVAR follow-up will likely require multicenter validation, standardized imaging protocols, and post-implementation performance monitoring over extended follow-up periods.

### 3.4. Peripheral Arterial Interventions

Peripheral arterial disease (PAD) is a significant cause of morbidity worldwide and frequently requires endovascular revascularization for symptomatic limb ischemia. Imaging plays a critical role in lesion detection, anatomical mapping, and procedure planning; methods such as duplex ultrasound, CTA, MRA, and DSA are used in this context. In this regard, AI-CAD systems aim to automate lesion identification, measure stenosis severity, and support procedure strategy selection.

#### 3.4.1. Lesion Detection on CTA and MRA

Automated detection of PAD in CTA and MRA has been investigated using deep learning-based vessel segmentation and lumen analysis frameworks. As with coronary CCTA, AI systems typically localize and grade stenoses by integrating vessel extraction, centerline tracking, and cross-sectional area measurement. While large-scale multicenter PAD-specific validation datasets remain limited, studies on automated vascular segmentation and stenosis assessment have demonstrated feasibility in retrospective cohorts [2].

However, peripheral artery imaging presents unique challenges compared to coronary or neurovascular regions. Lower extremity vessels are longer, more tortuous, and often affected by dense calcification, chronic total occlusion (CTO), and motion artifacts. These factors increase segmentation complexity and can reduce algorithm robustness, particularly in distal vessels.

#### 3.4.2. Angiographic Detection During Endovascular Procedures

Intra-procedural angiography remains the primary imaging method during peripheral interventions. AI-CAD systems applied to DSA have been investigated for automated stenosis localization, vessel diameter estimation, and flow assessment. Automated vessel segmentation in angiography has been studied in both coronary and peripheral contexts, with deep learning approaches demonstrating better reproducibility compared to manual measurements in controlled datasets [26].

From a bioengineering perspective, angiographic AI systems must address low contrast-to-noise ratios, overlapping vessel structures, patient movement, and dynamic contrast flow. Real-time implementation requires rapid inference and robustness against variable acquisition parameters. These engineering constraints are particularly important in peripheral interventions where image quality can vary significantly depending on lesion location and patient habitus.

#### 3.4.3. Chronic Total Occlusions and Complex Lesions

CTOs represent a technically challenging subset of peripheral interventions. Detection and characterization of CTO length, calcification burden, and collateral pathways influence device selection and crossing strategy. While AI applications specifically designed for peripheral CTO detection are relatively limited in the literature, vascular segmentation and plaque quantification frameworks developed for other vascular beds can be adapted to this environment [2]. From a technical standpoint, distinguishing a true chronic complete occlusion from the absence of opacification in a slow-filling vessel may require more than a single-frame lumen assessment. Multiphase or time-dependent angiographic analysis, collateral pathway assessment, and integration of proximal/distal vessel morphology can help artificial intelligence systems differentiate stable occlusion from delayed contrast passage [2,26].

Transitioning AI-CAD to peripheral interventions will rely on larger, annotated angiographic datasets and standardized reporting frameworks to ensure generalizable performance across various imaging environments.

#### 3.4.4. Translational Considerations in Peripheral AI-CAD

Compared to neurovascular and coronary fields, peripheral AI-CAD applications are in an earlier stage of development and are less represented among regulatory-cleared systems [10,11]. According to the evidence classification framework described in Section 2, many current tools fall under the scope of Tier 2 research prototypes or Tier 3 exploratory technologies.

Demonstrating clinical value in peripheral diseases may require proving not only high detection accuracy but also that AI integration improves procedural efficiency, reduces contrast use, or improves patency, limb salvage, and functional outcomes. Prospective, multicenter validation studies will be necessary to determine whether AI-CAD systems can meaningfully support decision-making in complex lower extremity interventions.

## 4. Evaluation and Validation of AI-CAD Systems in Endovascular Interventions

Robust evaluation is essential for the safe and effective transition of AI-CAD systems from retrospective development to clinical application. In endovascular interventions, the evaluation must account for high-consequence detection errors, heterogeneity of image acquisition, and workflow-oriented outcomes that extend beyond traditional measures of algorithmic accuracy. Accordingly, AI-CAD evaluation should be framed at three levels: (i) technical performance under controlled conditions, (ii) generalizability under real-world heterogeneity, and (iii) clinical impact when integrated into time-sensitive pathways.

**Figure 2:**
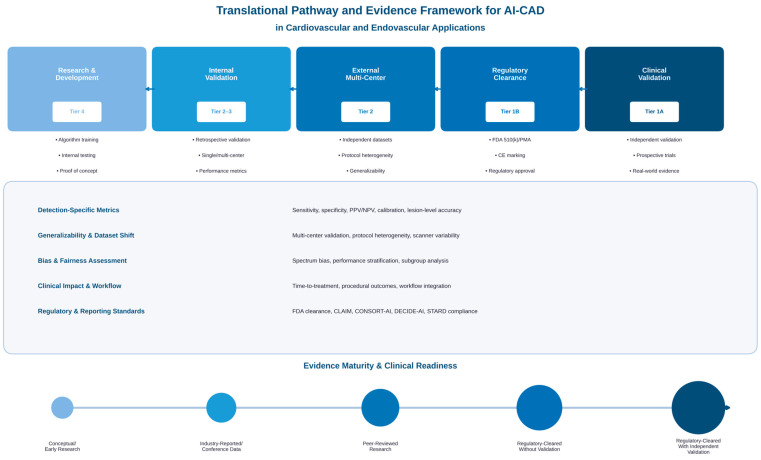
Translation path and evidence framework for AI-CAD in cardiovascular and endovascular applications. The figure illustrates the progression of AI-CAD systems from initial research and development (Tier 4) to internal validation (Tiers 2–3), external multicenter validation (Tier 2), regulatory clearance (Tier 1B), and clinical validation with independent evidence (Tier 1A). Five key areas of assessment are shown: (1) detection-specific metrics, including sensitivity, specificity, and calibration; (2) generalizability assessment across multiple centers and imaging protocols; (3) bias and fairness assessment through spectrum bias analysis and subgroup performance; (4) clinical impact measurement, including time to treatment initiation and procedural outcomes; and (5) regulatory and reporting standards in line with CLAIM, CONSORT-AI, DECIDE-AI, and STARD guidelines. The bottom-level evidence maturity scale demonstrates increasing evidence quality, from conceptual research to regulatory-cleared systems with independent clinical validation, by mapping the translation stages to the five-tier classification framework proposed in Table 1.

As shown in Figure 2, the successful implementation of AI-CAD requires going through structured validation phases accompanied by rigorous performance evaluation and bias assessment.

### 4.1. Detection-Specific Performance Metrics

AI-CAD evaluation requires careful alignment of metrics with the clinical detection task. For lesion-level detection issues, traditional discrimination metrics such as AUROC may be insufficient, as they can obscure the clinically significant balance between sensitivity and false positive burden. In detection settings, false positives can lead to alert fatigue or unnecessary subsequent imaging procedures, while false negatives can delay intervention. Therefore, performance reporting should include clinically relevant operating-point metrics reflecting how systems are used in practice, including analyses that clearly characterize sensitivity and false positive burden at a defined false positive rate.

For endovascular workflows, case-level and lesion-level evaluation should also be distinguished. Case-level evaluation may be suitable for triage tasks such as large vessel occlusion alerts, while lesion-level evaluation is more critical for localization of stenoses, identification of endoleaks, or detection of stent-related complications. Additionally, calibration should be reported when probabilistic outcomes affect clinical thresholds, as incorrect calibration can lead to inappropriate escalation or missed diagnoses despite high discrimination. Where possible, studies should also report confidence intervals and subgroup performance to indicate statistical and clinical uncertainty. This is particularly important in endovascular care because dose escalation decisions are often time-sensitive and may trigger irreversible downstream actions, such as urgent transfer, device preparation, additional angiography runs, or intraprocedural intervention. A poorly calibrated model may therefore lead to over-escalation despite acceptable discrimination, or under- escalation despite apparently strong overall accuracy [15,16,17].

Since many AI-CAD studies are structured as diagnostic accuracy studies, transparent reporting consistent with STARD 2015 principles supports interpretability and reproducibility, particularly in terms of patient selection, reference standards, and addressing ambiguous results [15].

### 4.2. Validation Strategies and Generalizability

A recurring hurdle to translation is limited external validation. Internal cross-validation can provide optimistic estimates when acquisition protocols, scanner features, or annotation practices are homogeneous. Temporal validation (training in earlier cohorts and testing in later cohorts) provides a pragmatic assessment of robustness against evolving clinical practice and technology. However, particularly in endovascular imaging where protocol heterogeneity and device-related artifacts are common, the most informative approach to assess generalizability is multicenter external validation.

In addition to field heterogeneity, AI-CAD systems can experience “dataset shift” related to population differences, prevalence variation, scanner manufacturers, reconstruction algorithms, or procedural differences. Therefore, validation should aim to test models under conditions that mimic application conditions, including variations in contrast bolus timing, metallic artifacts, and motion. For intra-procedural systems (e.g., angiography-based detection), latency and computational requirements become clinically relevant constraints and must be evaluated along with accuracy.

Importantly, the evaluation should reflect how AI-CAD is intended to function within the workflow. In neurovascular triage, improving time to treatment initiation may be a more clinically significant outcome than marginal changes in AUROC [12,13]. Similarly, intraoperative endoleak detection tools should be evaluated not only in terms of detection performance but also in terms of whether they alter intraoperative decision-making processes and reduce the need for re-intervention in later stages [14].

### 4.3. Bias, Fairness, and Quality of Evidence

Bias can enter the evaluation of AI-CAD through multiple mechanisms. Spectrum bias occurs when training or test datasets disproportionately represent severe disease or high-quality imaging, leading to inflated performance compared to routine practice. Label noise and inter-observer variability are particularly important in endovascular imaging, where reference standards may depend on expert interpretation and vary by institution. Additional risks include spurious correlations (shortcut learning), where models exploit confounding cues such as device appearance, acquisition cues, or institutional artifacts instead of the actual pathology.

Therefore, performance stratification among clinically relevant subgroups is important. Even if demographic fairness analysis is limited by sample size, reporting performance stratified by acquisition conditions (scanner type, contrast phase, artifact load) and clinical subtypes (e.g., endoleak type, lesion location, calcification severity) can reveal domain-specific weaknesses. In cases where predictive components are included (e.g., event risk models derived from imaging features), structured assessment tools such as PROBAST + AI can help assess the risk of bias and feasibility [27]. For detection-oriented systems, evidence classification frameworks, such as the stepwise approach used in this article, provide an additional layer of transparency by separating regulatory-cleared systems with independent validation from research prototypes.

### 4.4. Clinical Readiness, Study Design, and Reporting Standards

Clinical readiness for AI-CAD relies on more than just algorithmic performance. In endovascular pathways, the translation requires evidence of safe integration into human decision-making processes, a clear definition of intended use, and assessment designs that reflect real-world operation. Reporting standards can significantly improve transparency, comparability, and reproducibility. The CLAIM guideline for imaging-centric AI-CAD studies provide structured reporting elements for dataset description, reference standards, performance evaluation, and reproducibility considerations [16,28]. For clinical trials of AI interventions, CONSORT-AI improves reporting on how algorithms are integrated into trial workflows and how performance is monitored during evaluation [29]. Similarly, SPIRIT-AI provides guidance on protocol-level reporting to ensure methodological clarity before trial execution [30].

Between retrospective validation and large-scale randomized trials, early-stage clinical evaluation is increasingly considered a separate and necessary step, particularly to assess human factors, workflow integration, safety, and failure modes that only emerge in live environments. The DECIDE-AI guidance addresses this gap by outlining reporting expectations for early clinical evaluation of AI decision support systems, highlighting real-world use cases and human–AI interaction [17].

Taken together, these evaluation and reporting frameworks support a translational pathway where detection-driven metrics, external validation, bias-aware assessment, and workflow-centric clinical trials collectively define readiness for endovascular application. For AI-CAD systems designed for high-risk, time-critical decisions, the burden of proof should increasingly shift from retrospective performance to prospective demonstration of clinical utility and sustainable safety in routine practice. Based on the evaluation principles outlined above, Table 3 provides a structured checklist for assessing AI-CAD systems in endovascular applications; this checklist highlights detection-specific metrics, validation strategy, bias assessment, and regulatory context.

## 5. Translational Challenges and Future Directions

Despite the rapid proliferation of AI-CAD systems in endovascular settings, the transition from algorithmic promise to sustainable clinical impact is uneven. Previous sections have shown that detection performance in retrospective datasets often exceeds real-world reliability when confronted with imaging heterogeneity, workflow complexity, and human–AI interaction dynamics. Closing this translational gap requires coordinated advancements across technical development, validation methodology, regulatory science, and implementation strategy.

Table 4 details the key sources of dataset drift specific to endovascular imaging and suggests validation and mitigation strategies aligned with engineering and clinical requirements.

### 5.1. Closing the Performance-Impact Gap

A recurring theme in AI-CAD research is the divergence between high diagnostic discrimination and demonstrable clinical benefit. In endovascular contexts, marginal improvements in AUROC may not translate into meaningful patient-level outcomes unless detection outputs transform clinical decision-making. For example, improvements in treatment duration metrics in stroke networks have been associated with AI detection systems integrated into the workflow [12,13], highlighting that the system-level effect may be more significant than incremental accuracy gains.

Therefore, future studies should prioritize assessment designs that explicitly measure subsequent outcomes, including procedure timing, contrast utilization, re-intervention rates, and long-term device durability. Beyond detection, emerging AI systems are being investigated to support intravascular device selection and procedure strategy optimization in aortic disease [31], highlighting the broader decision support potential of AI in endovascular applications. Integrating AI-CAD into pragmatic clinical trials or registry-linked surveillance frameworks may provide more representative evidence than highly regulated retrospective validation cohorts.

### 5.2. Engineering Challenges in Heterogeneous Imaging Environments

Endovascular imaging is inherently heterogeneous. Variability in scanner hardware, reconstruction algorithms, contrast timing, metallic artifact load, and catheter movement leads to dataset shift that can degrade model performance outside of development environments. For intra-procedural systems, computational latency and real-time inference constraints add further complexity.

Emerging strategies to address these issues include domain adaptation techniques, continuous learning frameworks, and unified learning architectures that enable multi-center model refinement without centralized data transfer. However, these approaches introduce additional considerations related to data management, model drift monitoring, and regulatory oversight. To maintain trust in deployed systems, transparent reporting of acquisition conditions and failure cases will be critical.

### 5.3. Human–AI Interaction and Workflow Integration

AI-CAD systems function within socio-technical ecosystems, not as isolated algorithms. When sensing outputs are presented without proper contextualization, alert fatigue, overconfidence, and automation bias represent potential risks. Conversely, poorly integrated interfaces or delayed alerts can negate potential workflow benefits.

Future research should increasingly incorporate human factors assessment, usability testing, and simulation-based evaluation to characterize how clinicians interact with AI outputs. Reporting frameworks such as DECIDE-AI highlight the importance of examining not only accuracy but also adoption, interpretability, and failure mode awareness by emphasizing early-stage clinical evaluation under realistic workflow conditions [17].

### 5.4. Regulatory Evolution and Post-Market Surveillance

Regulatory oversight of AI-powered medical devices continues to evolve. Analyses of FDA-cleared AI/machine learning-powered devices are showing rapid growth in radiological fields, including cardiovascular imaging [10,11]. However, regulatory clearance primarily establishes safety and substantial equivalence within defined indications; it does not guarantee long-term performance stability under changing clinical conditions.

Post-market surveillance, performance monitoring, and transparent reporting of adverse events or deterioration in accuracy are increasingly recognized as key components of responsible AI use. For endovascular AI-CAD systems that can impact time-critical procedural decisions, structured post-use monitoring and the collection of real-world evidence are particularly important.

### 5.5. Towards Standardized Evidence Pathways

A recurring hurdle in the AI-CAD literature is heterogeneity in study design, performance metrics, and validation strategies. The adoption of structured reporting standards, including STARD, CLAIM, CONSORT-AI, SPIRIT-AI, and DECIDE-AI, can enhance transparency and comparability [15,16,17,29,30]. In parallel, pragmatic evidence classification frameworks, such as the tiered system proposed in this review, can help distinguish mature, independently validated systems from exploratory prototypes.

Future progress in endovascular AI-CAD will likely depend on harmonized evaluation criteria, multi-institutional collaboration, and the integration of engineering rigor with clinical realism. As AI technologies evolve toward basic models and multimodal architectures, maintaining a balance between technical innovation and clinical responsibility will be central to sustainable adoption.

## 6. Conclusions

AI-CAD systems are increasingly integrated into neurovascular, coronary, aortic, and peripheral endovascular fields. From large vessel occlusion triage to intravascular imaging support and endoleak detection, AI-CAD technologies demonstrate the potential to significantly improve diagnostic efficiency, procedure planning, and workflow coordination. However, algorithmic accuracy alone does not define clinical readiness.

This review highlights that meaningful use of AI-CAD in endovascular practice requires rigorous validation beyond retrospective performance metrics. Detection-specific evaluation, multicenter external validation, bias-aware assessment, and workflow-centric outcome studies are necessary to ensure robustness in heterogeneous imaging settings. Regulatory clearance, while a significant milestone, should be viewed not as definitive proof of sustainable efficacy in the real world, but as part of a broader continuum of evidence.

The tiered evidence classification framework proposed here offers a pragmatic approach to distinguish research prototypes from independently validated, regulatory-cleared systems. Structured reporting standards and post-implementation monitoring, along with such frameworks, can help align engineering innovation with clinical responsibility.

As endovascular imaging technologies continue to evolve and datasets expand, future AI-CAD systems will likely incorporate multimodal inputs, adaptive learning strategies, and tighter integration into procedural ecosystems. Ensuring these advancements translate into lasting improvements in patient outcomes will require ongoing collaboration among bioengineers, clinicians, regulatory scientists, and healthcare systems. Future research should focus on several critical directions to advance the safe use of AI-CAD technologies in endovascular care. First, multicenter prospective validation studies and registry-linked real-world evidence will be necessary to verify performance stability in heterogeneous imaging environments. Second, novel approaches such as federated learning and field adaptation can help mitigate dataset drift resulting from differences in scanner hardware, acquisition protocols, and patient populations. Third, integration of multimodal data, including imaging, procedural parameters, and electronic health records, can enable more comprehensive decision support systems extending beyond detection to procedure planning and risk prediction. Finally, as AI models evolve toward foundation models and multimodal architectures, transparency, explainability, and post-market monitoring will be critical to ensure that technical innovation translates into measurable improvements in patient outcomes.

Ultimately, the promise of AI-CAD in endovascular interventions lies not only in automated detection but also in responsible integration where technical excellence, transparent validation, and patient-centered evaluation come together to define clinical value.

## Figures and Tables

**Table 1:** Evidence classification framework for AI-CAD systems.

Tier	Description	Evidence Requirements	Limitations
1A	FDA/CE-cleared and peer-reviewed validation	Published clinical trials, regulatory clearance documentation, independent validation cohort	Highest level of confidence for clinical application
1B	FDA/CE-cleared without independent peer-reviewed validation	Regulatory clearance only, manufacturer-reported data	Clinical performance in real-world settings is uncertain
2	Peer-reviewed research without regulatory clearance	Published in indexed journals, academic validation	Not approved for clinical use
3	Industry-reported or conference presentations	Company-reported data, technical reports, conference abstracts	Unverified claims, potential publication bias
4	Conceptual/developmental	Pilot studies, prototypes, theoretical frameworks	Early-stage technologies

Note: This classification framework was developed for this review to systematically categorize the strength of evidence supporting AI-CAD systems in cardiovascular and endovascular applications. Tier assignment is based on regulatory status and the availability of independent peer-reviewed validation data.

**Table 2:** Representative AI-CAD systems in endovascular procedures according to field and translational status.

Domain	System/Example	Developer	Primary Modality	Primary Clinical Task	Regulatory Status (US/EU)	Regulatory Pathway/Identifier (If Known)	Evidence Tier	Notes/Typical Evidence Base
Neurovascular	Viz.ai LVO	Viz.ai	CTA	Large vessel occlusion detection/triage	US: cleared; EU: varies by module	De Novo and subsequent 510(k) modules (identifier to be specified)	1A	Prospective/real-world workflow studies and widespread application support the use of time-sensitive triage
Neurovascular	RapidAI stroke modules (e.g., LVO/ICH/CTP/ASPECTS) *	iSchemaView	NCCT/CTA/CTP	Stroke triage (ICH/LVO/CTP/ASPECTS depending on module)	US: cleared; EU: varies by module	510(k) modules (K numbers per module to be specified)	1A	Evidence is module-dependent; the strongest clinical value is generally demonstrated through workflow impact and time to treatment initiation outcomes
Coronary	HeartFlow FFR-CT	HeartFlow	CCTA	Functional lesion assessment (FFR-CT) supporting clinical decision-making processes	US: cleared/authorized; EU: varies	De Novo with subsequent 510(k) updates (identifier to be specified)	1A	Large clinical evidence base should be included (physiology modeling rather than pure “lesion detection”) as a translationally mature coronary AI platform
Coronary	AI-enabled plaque analysis (e.g., HeartFlow Plaque Analysis or equivalent) **	HeartFlow/others	CCTA (±IVUS reference in studies)	Plaque feature detection/quantification supporting risk discussion and planning	US: cleared for specific indications (module specific)	510(k) (identifier to be specified if used)	1B-1A	Evidence strength varies depending on the module and the availability of independent validation; specify the full module if it is to be maintained
Aortic/EVAR	Endoleak detection in CTA (research prototypes)	Academic groups	CTA	Endoleak detection and/or classification; sac monitoring support	Not cleared (typical)	-	2	Generally retrospective single-center studies; external validation under protocol variability and metallic artifacts is of great importance.
Aortic/EVAR	In-procedure endoleak detection	Academic/early translational	DSA	Endoleak detection/localization during EVAR completion angiography	Not cleared (typical)	-	2	Early feasibility; clinically important but requires prospective evaluation for procedural decision impact
Peripheral	PAD lesion detection/stenosis grading (research)	Academic/early translational	CTA/DSA	Stenosis detection and grading; runoff assessment	Not cleared (typical)	-	2–3	Evidence is often limited by the dataset; generalizability challenges exist due to calcification, long vessel segments, motion, and protocol heterogeneity

* RapidAI (iSchemaView) is a modular platform comprising multiple individually cleared 510(k) modules (e.g., LVO, ICH, CTP, ASPECTS). Evidence strength, clinical utility, and regulatory pathway details vary by module; specific 510(k) K-numbers should be confirmed with the manufacturer for each module referenced. ** AI-enabled plaque analysis platforms (e.g., HeartFlow Plaque Analysis) may encompass multiple sub-modules with varying levels of independent validation. The evidence tier assigned (1B–1A) reflects this heterogeneity; the specific module and its corresponding validation status should be specified when referencing these systems in clinical or regulatory contexts.

**Table 3:** Evaluation and validation framework of AI-CAD systems in endovascular procedures.

Domain	Key Considerations	Recommended Reporting Elements	Rationale for Endovascular Context
Detection task definition	Clear definition of detection target (e.g., LVO, endoleak, stenosis ≥ 50%, stent malapposition)	Explicit lesion criteria; reference standard description; handling of equivocal cases	Ambiguous target definitions inflate performance estimates and reduce comparability
Performance metrics	Appropriate metrics aligned with the detection task	Sensitivity, specificity, PPV/NPV; sensitivity at fixed FP rate; lesion-level vs. case-level analysis; confidence intervals	AUROC alone can mask a clinically significant false positive burden
Calibration	Reliability of predicted probabilities	Calibration plots; Brier score; threshold justification	Miscalibration can affect clinical triage thresholds and escalation decisions
Internal validation	Prevention of overfitting	Cross-validation or hold-out testing; separation of training and test datasets	Reduces optimism bias in homogeneous datasets
External validation	Generalizability across centers	Independent multi-center validation; reporting of scanner vendors and protocols	Endovascular imaging is highly heterogeneous across institutions
Dataset shift assessment	Robustness against protocol variability	Evaluation across contrast phases, artifact burden, acquisition parameters	Contrast timing and metallic artifacts significantly affect detection
Subgroup analysis	Performance consistency	Stratified results by lesion type, size, location, imaging quality	Small lesions or distal vessels often reduce detection accuracy
Workflow integration	Real-world deployment	Description of alert timing, latency, user interface, human–AI interaction	In stroke and intra-procedural EVAR, timing is clinically critical
Clinical impact	Beyond diagnostic accuracy	Time-to-treatment metrics; change in procedural strategy; reintervention rates	Workflow benefit may exceed marginal changes in AUROC
Bias and fairness	Spectrum and selection bias	Description of dataset composition; inclusion/exclusion criteria; annotation process	Training on severe or high-quality cases inflates performance
Reproducibility and transparency	Reporting standards	Adherence to STARD/CLAIM; code/model availability where feasible	Enhances scientific transparency and regulatory confidence
Regulatory status	Translational maturity	FDA clearance pathway (510(k), De Novo, PMA); post-market monitoring if available	Regulatory clearance is not the same as external clinical validation

**Table 4:** Common sources of dataset shift in endovascular imaging and mitigation strategies for AI-CAD systems.

Source of Dataset Shift	Typical Context in Endovascular Imaging	Potential Impact on AI-CAD Performance	Proposed Validation Strategy	Potential Mitigation Approaches
Contrast timing variability	CTA multiphase protocols in stroke, CCTA timing differences, delayed EVAR phase acquisition	Reduced sensitivity for low-contrast lesions (e.g., endoleaks), misclassification of stenosis severity	External validation in various contrast protocols; phase-specific subgroup analysis	Protocol-aware training; phase normalization; inclusion of multi-phase training data
Metallic artifacts	Stent grafts (EVAR), coronary stents, dense calcification in PAD	False positives around metal; segmentation instability; attenuation misestimation	Strategic performance reporting in metal-heavy cases	Metal artifact reduction preprocessing; artifact-robust augmentation; uncertainty prediction flags
Scanner vendor variability	Different CT manufacturers and reconstruction cores	Domain shift affecting tissue-based features and radiomics	Multiple vendor external validation; vendor stratified reporting	Domain adaptation; vendor-balanced training datasets
Reconstruction algorithms	Iterative reconstruction vs. filtered back projection; varying slice thickness	Altered image texture affecting learned feature maps	Temporal validation in software updates	Image harmonization; training on mixed reconstruction types
Motion artifacts	Cardiac motion (CCTA), respiratory motion (aortic imaging), patient motion in DSA	Reduced lesion detectability; segmentation failure	Subgroup analysis based on image quality score	Motion-robust architectures; quality control modules
Rare lesion prevalence	Type II endoleaks, distal PAD lesions, small aneurysms	Inflated AUROC in enriched datasets; decreased PPV in real-world prevalence	Prevalence-adjusted assessment; PPV/NPV reporting at realistic disease rates	Balanced sampling; cost-sensitive learning; calibration adjustment
Labeling variability	Inter-observer discrepancy in IVUS/OCT or subtle endoleak	Label noise; decreased reproducibility	Multiple reader reference standards; inter-reader reliability reporting	Consensus labeling; probabilistic labeling; noise-robust loss functions
Temporal application evolution	Changes in devices, techniques, and imaging protocols over time	Post-distribution model distortion (model drift)	Temporal validation; post-market monitoring	Continuous learning with safeguards; periodic revalidation

## Data Availability

The datasets supporting the findings of this review are cited within the article and are available in the referenced original publications.

## References

[1] World Health Organization (2025). Cardiovascular Diseases (CVD). https://www.who.int/news-room/fact-sheets/detail/cardiovascular-diseases-(cvds).

[2] Wang Z., Yi R., Wen X., Zhu C., Xu K. (2024). Cardiovascular medical image and analysis based on 3D vision: A comprehensive survey. Meta-Radiol..

[3] Singh H., Meyer A.N., Thomas E.J. (2014). The frequency of diagnostic errors in outpatient care: Estimations from three large observational studies involving US adult populations. BMJ Qual. Saf..

[4] Balogh E.P., Miller B.T., Ball J.R., Committee on Diagnostic Error in Health Care, Board on Health Care Services, Institute of Medicine (2015). Improving Diagnosis in Health Care.

[5] Lin M.P., Burke R.C., Sabbatini A.K., Latsko E., Edlow J.A., Orav E.J., Burke L.G. (2025). Potential Diagnostic Error for Emergency Conditions, Mortality, and Healthy Days at Home. JAMA Netw. Open.

[6] Saver J.L. (2006). Time is brain--quantified. Stroke.

[7] Ardic N., Dinc R. (2025). Artificial Intelligence in Healthcare: Current Regulatory Landscape and Future Directions. Br. J. Hosp. Med..

[8] U.S. Food and Drug Administration (FDA) (2025). Artificial Intelligence-Enabled Medical Devices. https://www.fda.gov/medical-devices/software-medical-device-samd/artificial-intelligence-enabled-medical-devices.

[9] Willemink M.J., Roth H.R., Sandfort V. (2022). Toward Foundational Deep Learning Models for Medical Imaging in the New Era of Transformer Networks. Radiol. Artif. Intell..

[10] Sivakumar R., Lue B., Kundu S. (2025). FDA Approval of Artificial Intelligence and Machine Learning Devices in Radiology: A Systematic Review. JAMA Netw. Open.

[11] Sardar P., Dudorova E., Chatterjee S., Parikh S.A., Brilakis E.S., Singh J.P. (2025). Analysis of FDA-Approved Artificial Intelligence and Machine Learning-Enabled Cardiovascular Devices. JACC Adv..

[12] Martinez-Gutierrez J.C., Kim Y., Salazar-Marioni S., Tariq M.B., Abdelkhaleq R., Niktabe A., Ballekere A.N., Iyyangar A.S., Le M., Azeem H. (2023). Automated Large Vessel Occlusion Detection Software and Thrombectomy Treatment Times: A Cluster Randomized Clinical Trial. JAMA Neurol..

[13] Le N.M., Iyyangar A.S., Kim Y., Chaudhry M.R., Salazar-Marioni S., Abdelkhaleq R., Niktabe A., Ballekere A.N., Azeem H., Shaw S. (2024). Machine Learning-Enabled Automated Large Vessel Occlusion Detection Improves Transfer Times at Primary Stroke Centers. Stroke Vasc. Interv. Neurol..

[14] Smorenburg S.P.M., Hoksbergen A.W.J., Yeung K.K., Wolterink J.M. (2025). Multitask Deep Learning for Automated Detection of Endoleak at Digital Subtraction Angiography during Endovascular Aneurysm Repair. Radiol. Artif. Intell..

[15] Bossuyt P.M., Reitsma J.B., Bruns D.E., A Gatsonis C., Glasziou P.P., Irwig L., Lijmer J.G., Moher D., Rennie D., de Vet H.C.W. (2015). STARD 2015: An Updated List of Essential Items for Reporting Diagnostic Accuracy Studies. Clin. Chem..

[16] Mongan J., Moy L., Kahn C.E. (2020). Checklist for Artificial Intelligence in Medical Imaging (CLAIM): A Guide for Authors and Reviewers. Radiol. Artif. Intell..

[17] Vasey B., Nagendran M., Campbell B., Clifton D.A., Collins G.S., Denaxas S., Denniston A.K., Faes L., Geerts B., Ibrahim M. (2022). Reporting guideline for the early-stage clinical evaluation of decision support systems driven by artificial intelligence: DECIDE-AI. Nat. Med..

[18] Griffin W.F., Choi A.D., Riess J.S., Marques H., Chang H.-J., Choi J.H., Doh J.-H., Her A.-Y., Koo B.-K., Nam C.-W. (2023). AI Evaluation of Stenosis on Coronary CTA, Comparison With Quantitative Coronary Angiography and Fractional Flow Reserve: A CREDENCE Trial Substudy. JACC Cardiovasc. Imaging.

[19] Nurmohamed N.S., Danad I., Jukema R.A., de Winter R.W., de Groot R.J., Driessen R.S., Bom M.J., van Diemen P., Pontone G., Andreini D. (2024). Development and Validation of a Quantitative Coronary CT Angiography Model for Diagnosis of Vessel-Specific Coronary Ischemia. JACC Cardiovasc. Imaging.

[20] Lin A., Manral N., McElhinney P., Killekar A., Matsumoto H., Kwiecinski J., Pieszko K., Razipour A., Grodecki K., Park C. (2022). Deep learning-enabled coronary CT angiography for plaque and stenosis quantification and cardiac risk prediction: An international multicentre study. Lancet Digit. Health.

[21] Ihdayhid A.R., Sehly A., He A., Joyner J., Flack J., Konstantopoulos J., Newby D.E., Williams M.C., Ko B.S., Chow B.J. (2024). Coronary Artery Stenosis and High-Risk Plaque Assessed With an Unsupervised Fully Automated Deep Learning Technique. JACC Adv..

[22] Pociask E., Malinowski K.P., Ślęzak M., Jaworek-Korjakowska J., Wojakowski W., Roleder T. (2018). Fully Automated Lumen Segmentation Method for Intracoronary Optical Coherence Tomography. J. Healthc. Eng..

[23] Lee J., Prabhu D., Kolluru C., Gharaibeh Y., Zimin V.N., Dallan L.A.P., Bezerra H.G., Wilson D.L. (2020). Fully automated plaque characterization in intravascular OCT images using hybrid convolutional and lumen morphology features. Sci. Rep..

[24] Lee J., Gharaibeh Y., Kolluru C., Zimin V.N., Dallan L.A.P., Kim J.N., Bezerra H.G., Wilson D.L. (2020). Segmentation of Coronary Calcified Plaque in Intravascular OCT Images Using a Two-Step Deep Learning Approach. IEEE Access.

[25] Chaikof E.L., Dalman R.L., Eskandari M.K., Jackson B.M., Lee W.A., Mansour M.A., Mastracci T.M., Mell M., Murad M.H., Nguyen L.L. (2018). The Society for Vascular Surgery practice guidelines on the care of patients with an abdominal aortic aneurysm. J. Vasc. Surg..

[26] Moccia S., De Momi E., El Hadji S., Mattos L.S. (2018). Blood vessel segmentation algorithms—Review of methods, datasets and evaluation metrics. Comput. Methods Programs Biomed..

[27] Moons K.G.M., Damen J.A.A., Kaul T., Hooft L., Navarro C.A., Dhiman P., Beam A.L., Van Calster B., Celi L.A., Denaxas S. (2025). PROBAST+AI: An updated quality, risk of bias, and applicability assessment tool for prediction models using regression or artificial intelligence methods. BMJ.

[28] Tejani A.S., Klontzas M.E., Gatti A.A., Mongan J.T., Moy L., Park S.H., Kahn C.E., Panel F.T.C.2.U., Abbara S., Afat S. (2024). Checklist for Artificial Intelligence in Medical Imaging (CLAIM): 2024 Update. Radiol. Artif. Intell..

[29] Liu X., Rivera S.C., Moher D., Calvert M.J., Denniston A.K., SPIRIT-AI and CONSORT-AI Working Group (2020). Reporting guidelines for clinical trial reports for interventions involving artificial intelligence: The CONSORT-AI Extension. BMJ.

[30] Rivera S.C., Liu X., Chan A.W., Denniston A.K., Calvert M.J., SPIRIT-AI and CONSORT-AI Working Group (2020). Guidelines for clinical trial protocols for interventions involving artificial intelligence: The SPIRIT-AI Extension. BMJ.

[31] Dinc R., Ardic N. (2025). AI-driven decision making for intravascular device selection in aortic disease. Current insights and prospects. Front. Cardiovasc. Med..

